# Nutrigenomic Effects of Long-Term Grape Pomace Supplementation in Dairy Cows

**DOI:** 10.3390/ani10040714

**Published:** 2020-04-19

**Authors:** Marianna Pauletto, Ramy Elgendy, Andrea Ianni, Elettra Marone, Mery Giantin, Lisa Grotta, Solange Ramazzotti, Francesca Bennato, Mauro Dacasto, Giuseppe Martino

**Affiliations:** 1 Department of Comparative Biomedicine and Food Science, University of Padua, Viale dell’Università 16, 35020 Legnaro (PD), Italy; marianna.pauletto@unipd.it (M.P.); ramy.elgendy@igp.uu.se (R.E.); mery.giantin@unipd.it (M.G.); 2 Department of Pharmacology, Faculty of Veterinary Medicine, Suez Canal University, Ismailia 41522, Egypt; 3 Faculty of Bioscience and Technology for Food, Agriculture and Environment, University of Teramo, 64100 Teramo (TE), Italy; aianni@unite.it (A.I.); emarone@unite.it (E.M.); lgrotta@unite.it (L.G.); sramazzotti@unite.it (S.R.); fbennato@unite.it (F.B.); gmartino@unite.it (G.M.)

**Keywords:** nutrigenomics, grape pomace, polyphenols, dairy cows, RNA-sequencing

## Abstract

**Simple Summary:**

The aim of this study was to evaluate the effect of grape pomace (GP), the polyphenol-rich agricultural by-product, on dairy cows’ whole-blood transcriptome, milk production and composition. Twelve lactating Holstein-Friesian cows were randomly assigned to two groups; the first received a GP-supplemented diet for 60 days (group GP), whereas the second was given only a basal diet (CTR). The results reveal 40 protein-coding genes differentially expressed in the GP group when compared with the CTR group, but no effects were noticed on milk production, concentrations of crude protein, fat, casein, lactose and urea, or somatic cell count. Compared to CTR, GP had a transcriptomic signature mainly reflecting a reinforced immunogenic response.

**Abstract:**

The increasing demand for more animal products put pressure on improving livestock production efficiency and sustainability. In this context, advanced animal nutrition studies appear indispensable. Here, the effect of grape pomace (GP), the polyphenol-rich agricultural by-product, was evaluated on Holstein-Friesian cows’ whole-blood transcriptome, milk production and composition. Two experimental groups were set up. The first one received a basal diet and served as a control, while the second one received a 7.5% GP-supplemented diet for a total of 60 days. Milk production and composition were not different between the group; however, the transcriptome analysis revealed a total of 40 genes significantly affected by GP supplementation. Among the most interesting down-regulated genes, we found the DnaJ heat-shock protein family member A1 (DNAJA1), the mitochondrial fission factor (MFF), and the impact RWD domain protein (IMPACT) genes. The gene set enrichment analysis evidenced the positive enrichment of ‘interferon alpha (IFN-α) and IFN-γ response’, ‘IL6-JAK-STAT3 signaling’ and ‘complement’ genes. Moreover, the functional analysis denoted positive enrichment of the ‘response to protozoan’ and ‘negative regulation of viral genome replication’ biological processes. Our data provide an overall view of the blood transcriptomic signature after a 60-day GP supplementation in dairy cows which mainly reflects a GP-induced immunomodulatory effect.

## 1. Introduction

Historically, human populations largely depend on domestic farm animals for the production of animal food-products (e.g., meat, fat, milk and other dairy products). The recent and increasing demand for more animal products, especially in developing countries with rising living standards, puts pressure on global livestock productivity. With a rapidly growing world population, this increasing demand will put a strain on our natural resources and the whole environment as livestock directly compete with humans for the available arable land [1]. Nonetheless, the high demand for these animal proteins has to deal with other challenges such as climate change. To improve further animal production efficiency and limit the global land use for livestock production, advanced animal nutrition studies are today indispensable. 

In this context, a thorough understanding of animal gene expression driven by dietary nutrients can be regarded as the bottom line of advanced animal nutrition research. Nutrigenomics (including transcriptomics) is a new branch of animal nutrition at the basic molecular biological level. In other words, it studies the effects of dietary nutrients on cellular gene expression and ultimately, phenotypic changes in living animals. Among the transcriptomic approaches, RNA-sequencing (RNA-seq) simultaneously measures the differential expression of thousands of genes in any biological matrix. The resulting information can be used to determine the effects of a certain condition (e.g., a supplemented diet) from the perspective of the biological processes or molecular functions involved rather than from the expression levels of individual genes [2].

In the European Union (EU), agro-industrial by-products are being considered a key element in the EU’s 2020 Environment Action Programme concerning waste management, recycling, and reuse [3]. The reuse of agricultural by-products to produce bio-liquids, oils [4] and bioactive compounds such as carotenoids and polyphenols [5,6] or as animal feed and land fertilizers [5,6,7], has valorized them as sustainable resources. Indeed, the use of several agricultural by-products in animal nutrition has been largely explored [7]. In addition to the ‘traditional’ agricultural residues such as oil meals, bran, brewers’ grains, beet pulp and molasses, other ‘alternative’ by-product residues, resulting from fruit and vegetable processing, have become attractive as animal feedstuffs [8].

Grape pomace (GP) is the residue (by-product) that remains after crushing the grapes to collect its juice. It makes about one third of the total volume of the grapes used in wine production [9]. Although biodegradable, GP requires time to mineralize; thus, if left unexploited, it might represent a potential source of pollution to the environment [10]. However, GP is rich in phenolic compounds [9] that hold substantial antioxidant properties [11,12]. Moreover, due to its low cost and high fiber content, GP was proposed earlier as an alternative feed ingredient to ‘partially’ replace the forage portion in the diet of ruminants [9]. 

Recently, there has been growing interest in studying the effects of the dietary inclusion of GP on the overall productive performances and production aspects of food-producing animals. For instance, GP increased the biodiversity degree of intestinal microflora in broiler chicks [13], and improved the gain-to-feed ratio and overall performance in pigs [14,15]. Further, GP altered the nitrogen metabolism and decreased the ruminal ammonia production in male sheep [16], was shown to modify the rumen microbial population involved in methane metabolism [17], enhance the growth of facultative probiotic bacteria and inhibit the growth of pathogenic ones in lambs [18], but had no effect on milk yield and composition of dairy ewes [19]. In dairy cows, few studies, with varying results, have investigated the effects of GP dietary inclusion on milk production, composition and total polyphenols content as well as on the overall performance of animals [10,20,21,22,23]. We have previously described the transcriptomic signature of veal calves fed with a GP-supplemented diet [24]. Nevertheless, to our knowledge, few papers concerning the nutrigenomic effect of long-term dietary GP supplementation in dairy cows are available. It is conceivable that the dietary inclusion of GP (5% of total diet) would have a noticeable transcriptomic signature that could reflect its claimed antioxidant effects in dairy cows. Therefore, in the present study, a whole-transcriptome profiling, using the RNA-seq technology, was performed in dairy cows fed with a GP-supplemented diet and the obtained results are described herein.

## 2. Materials and Methods

### 2.1. Animals and Study Design

Animals were managed as per the Directive 2010/63 [25] of the European Parliament regarding the protection of animals used for experimentation or other scientific purposes. Blood sampling was performed concurrently with planned blood withdrawal for prophylaxis. Dairy cows had not been subjected to breeding practices different than those normally envisaged; thus, ethical declaration was not necessary.

Twelve lactating Holstein-Friesian cows (~163 days in milk), with an average calving number of 2.55 and an average body condition score of 2.97 were used as experimental animals for this study. The cows had an acclimation period of 2 weeks; then, they were randomly allocated into 2 experimental groups of 6 animals each. The first group received a basal diet and served as a control (CTR), while the other group received a diet supplemented with 7.5% GP-supplemented diet on a dry matter basis (GP group). The experimentation was conducted for a period of 60 days, in which all animals were housed in 2 separate areas of free housing with access to an identical feeding area in which each animal had an individual feeding bin, with water freely available all throughout the study. The GP, obtained from different wineries and distilleries in the region of Abruzzo (Italy), was prepared according to the procedure described in [26], and analyzed for its chemical composition before being added to the animals’ diet. Weekly from the start of the trial, the daily feed intake (dry matter) has been calculated for each animal by subtracting the refused meal to the administered diet.

### 2.2. Extraction and Determination of GP Total Polyphenols Content (TPC)

In order to extract the GP polyphenols, 1 g of pomace was added to 5 mL of methanol and the mixture was stirred for 4 h at room temperature. The solution was then transferred in a 15 mL test tube and placed in an ultrasonic bath for 10 min. At the end of this procedure, centrifugation (4000 rpm) was carried out for 20 min. The supernatant was recovered and filtered with paper filters (Whatman 41) in order to remove the interfering substances; this operation was repeated three times. Total Phenolic Content (TPC) was estimated by using the Folin-Ciocalteu colorimetric method, according to the procedure reported by [27]. The chemical composition and total polyphenols content of the GP extract are reported in Table 1.

### 2.3. Sampling

Milk, feed, and whole-blood (WB) samples were individually collected from each group. For the RNA-seq analysis, 2.5 mL of jugular venous blood were collected at T60 (2 groups × 6 animals each). Duplicate WB samples were collected in PAXgene^TM^ tubes (Qiagen SpA, Milan, Italy), stored at room temperature overnight and, then, at −20 °C until RNA isolation. Milk yield (morning milking) was recorded for each cow and individual milk samples were collected from all animals and stored at −20 °C for further analysis.

### 2.4. Milk and Feed Composition Analysis

Milk samples were analyzed for their protein, casein, lactose, fat, urea, pH, and total solids content (%) using Fourier Transformed InfraRed (FTIR) spectrophotometry (MilkoScanTM FT 6000; Foss, Hillerød, Denmark) as per the manufacturer’s instructions. The milk somatic cell count (SCC) was analyzed by a fluoro-opto-electronic counter (FossomaticTM FC; Foss, Hillerød, Denmark). The method is based on flow cytometry technology that counts somatic cells, following the mixing of milk samples (1 to 10 µL, at 30–42 °C) with a DNA molecule-binding staining solution. The administered diets were analyzed according to the methods described by AOAC International [28] in order to obtain information about dry matter (method 930.15), crude protein (method 954.01), fat content (method 920.39), crude fibre (method 962.09) and ash (method 942.05). The procedure reported by Goering and Van Soest [29] was instead used for the evaluation of Neutral Detergent Fibre (NDF) and Acid Detergent Fibre (ADF). The composition of the basal and supplemental diets is reported in Table 2.

### 2.5. Library Preparation and RNA-Seq Analysis

Total RNA was isolated from blood samples by using the PAXgene blood RNA kit (Qiagen, Milan, Italy) as per the manufacturer’s instructions. To reduce the possible presence of genomic DNA contamination, a 15-minutes on-column DNase digestion step was included in the RNA isolation protocol. Total RNA concentration was determined by the NanoDrop ND-1000 spectrophotometer (NanoDrop Technologies Inc., Wilmington, DE, USA), and its quality was measured by the 2100 Bioanalyzer and RNA 6000 Nano kit (Agilent Technologies, Santa Clara, CA, USA). Strand-specific RNA-seq libraries were prepared using the SureSelect strand-specific mRNA library preparation kit (Agilent Technologies, Santa Clara, CA, USA) as per the manufacturer’s protocol. In brief, poly(A) RNA was purified from 1 µg of total RNA using two serial rounds of binding to oligo(dT) magnetic particles; then, the nucleic acid was fragmented and reverse transcribed to generate cDNA. Illumina-specific adaptor was sequentially ligated to the 3’ end of cDNA fragments, purified using the AMPure XP beads (Beckman Coulter, Brea, CA, USA) and, finally, PCR-amplified (13 cycles) using an appropriate indexing primer to allow further samples multiplexing. The PCR-amplified libraries were purified with the AMPure XP beads (Beckman Coulter, Brea, CA, USA) and then assessed for their quality and fragments distribution using the 2100 Bioanalyzer DNA 1000 assay (Agilent Technologies, Santa Clara, CA, USA). In the presence of adaptor-dimers (electropherogram’s peak at 100 to 150-bp), another round of magnetic beads purification was performed. Libraries were quantified by both the Qubit® Fluorometer (Life Technologies, Monza, Italy) and the qPCR-based NEBNext library quantification kit (New England BioLabs, Hitchin, UK). Finally, equimolar amounts of each 6 index-tagged libraries were multiplexed together in one pool (total of 4 pools, 24 single libraries) and then sequenced by an Illumina HiSeq 2500 for 50 sequencing cycles.

The raw 50 bp single-end sequences (Sanger/Illumina 1.9 encoding) were quality-controlled using FastQC v.0.11.4 [30], and the low-quality bases (Phred quality score <30) and adaptor contamination (if present) were removed by Trimmomatic v.0.36 [31], using the parameters ‘LEADING:3 SLIDINGWINDOW:4:20 MINLEN:25’. The high-quality reads were mapped by HISAT v. 2.0.4 [32] against the *Bos taurus* reference genome (Ensembl UMD 3.1). The uniquely-mapped reads aligned to exons were counted with HTSeq v.0.6.1 [33], then tested—by the DESeq2 R package v.1.14.1 [34]—for the presence of differentially expressed genes (DEGs) between CTR and GP groups after the 60-day supplementation period. All genes with a false discovery rate (FDR) less than 0.1 were considered DEGs regardless of their fold-change (FC) value. The sequencing data (FASTQ files) associated with this project are deposited in the GenBank’s Sequence Read Archive (SRA) under the accession number SRP105401.

### 2.6. Functional Analysis and Statistics

The Gene Set Enrichment Analysis (GSEA) was used to examine the significantly enriched pathways in the GP group—in comparison with the CTR group. GSEA is a computational method that identifies shared differential gene expression of predefined, functionally related gene sets representing biological pathways. This is quantified by using a different type of Enrichment Score (ES), a weighted Kolmogorov–Smirnov-like statistic that evaluates if the members of the pathway are randomly distributed or found at the extremes (top or bottom) of the list [35]. In the present study, the GSEA pre-ranked option was used to analyze the deregulated pathways in the GP-supplemented group (GP T60 vs. CTR T60). All the Ensembl gene IDs were collapsed to their corresponding HUGO gene symbols; then, the entire normalized transcriptome dataset was ranked by the logarithm transformed (base 2) FC, where the up- and down-regulated genes were assigned positive and negative values, respectively. The pre-ranked dataset was analyzed (1000 permutations) against the Hallmarks (h.all.v7.0), i.e., the curated canonical KEGG pathways (c2.cp.kegg.v7.0.) and the gene ontology biological process (c5.bp.v7.0) catalogs from the Molecular Signatures Database (MsigDB) [35]. The GSEA output reported in the present study represents the most negatively- or positively-enriched hallmarks, pathways or biological processes (highest normalized enrichment score; NES) in the GP group compared with CTR. To visualize the gene expression data of GP and CTR samples, principal component analysis (PCA) and hierarchical clustering plots were generated using the ClustVis R package [36].

## 3. Results

The health status of all the experimental animals was satisfactory all throughout the experiment, with no visible signs of illness or supplementation-related stress. The dairy cows produced an average daily amount of 17 ± 2.4 liters of milk. The effect of the GP supplementation on the daily amount of milk produced as well as on the milk biochemical profile is shown in Supplementary Figure S1. Long-term GP supplementation did not result in differences between the GP and CTR groups either in the daily amount of milk produced than in milk’s biochemical profile, i.e., the percentage of fat, protein, casein, lactose, urea and solids. In addition to this, and in strict accordance with what was observed for the milk yield, no significant variations were highlighted in feed intake for the entire duration of the trial, with a daily dry matter intake (DMI) equal to 19.3 ± 1.2 and 19.5 ± 0.9 kg in CTR and GP group respectively.

**Figure 1:**
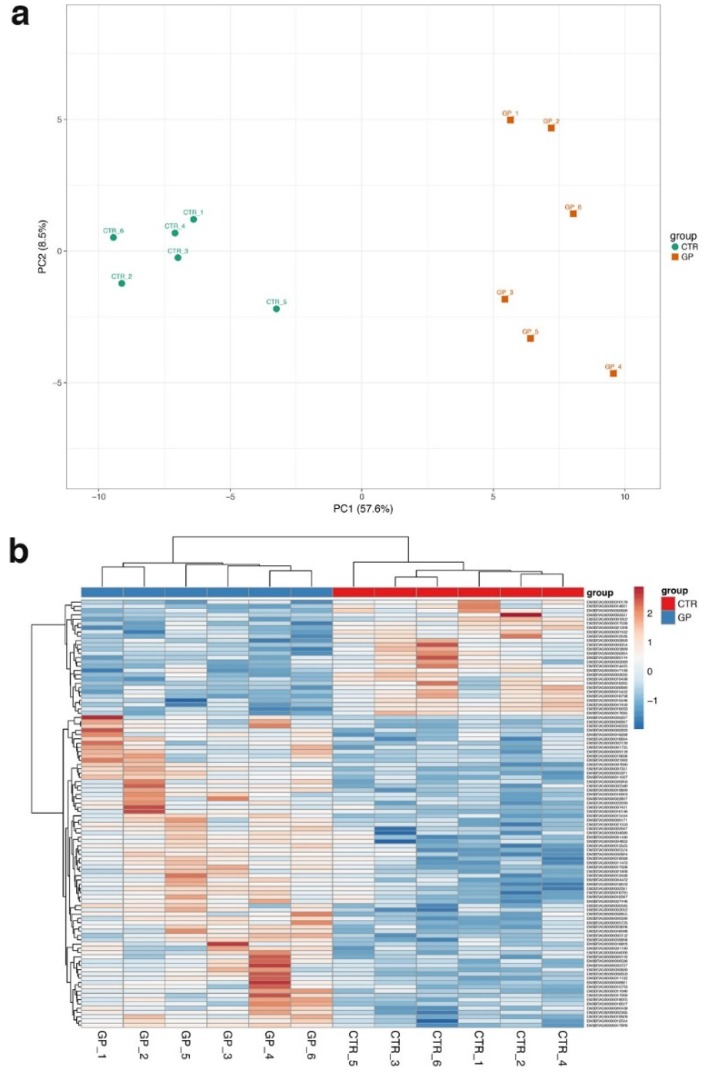
Principal Component Analysis (PCA) and hierarchical clustering heatmap. PCA and heatmap of the top 100 deregulated genes in dairy cows fed for 60 days with a GP-supplemented diet (GP T60 vs. CTR T60). (**a**) PCA plot of the GP (orange squares, right side) and CTR individuals (green circles, left side) after 60 days of GP dietary supplementation. (**b**) Heatmap generated from the DEGs using the hierarchical clustering (Euclidean distance clustering algorithm) of the top 100 deregulated genes of the GP group.

Regarding RNA-seq, the sequencing of 12 samples (2 groups × 6 animals/group) resulted in an average of~21 million reads per sample. A summary of the sequencing output is reported in Supplementary Table S1. After the assumption of a GP-supplemented diet for 60 days, 40 protein-coding genes were found to be differentially expressed in the GP group, of which 10 and 30 genes were down- and up-regulated, respectively (Supplementary Table S2). The most down-regulated gene in the GP group (FDR < 0.05) was the DnaJ heat shock protein family (HSP40) member A1. Additional interesting genes down-regulated by GP supplementation were the mitochondrial fission factor (MFF), the impact RWD domain protein (IMPACT), the ATP binding cassette subfamily A member 13 (ABCA13), the regulator of G-protein signaling 2 (RGS2), and the integrin subunit alpha 3 (ITGA3). The top 100 deregulated genes (using their normalized RNA-seq counts) were able to discriminate the two experimental groups either through a principal component analysis (PCA) plot (with the first 2 components accounting for > 60% of the total variability; Figure 1a) or by a hierarchical clustering heatmap (Figure 1b).

**Figure 2:**
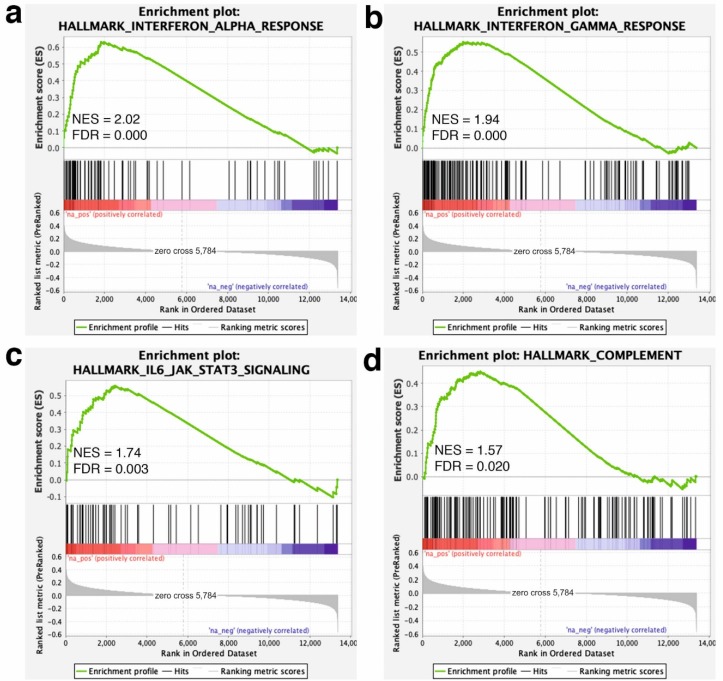
Gene set enrichment analysis (GSEA) plot (score curves) for enriched hallmarks. The GSEA analysis was performed with the hallmarks gene sets of GSEA Molecular Signatures Database. The “Signal-to-Noise” ratio (SNR) statistic was used to rank the genes per their correlation with the dietary GP supplementation (red) or the un-supplemented CTR phenotype (blue). The heatmap on the right side of each panel visualize the genes mostly contributing to the enriched gene set. For the fully detailed list, see Supplementary Table S3. The green curve corresponds to the Enrichment Score (ES) curve, which is the running sum of the weighed ES obtained from the GSEA software, while the Normalized Enrichment Score (NES) and the corresponding *p*-value are reported within each graph. Panels (**a**–**d**) denote the most enriched (significant) pathways (i.e., gene sets).

**Figure 3:**
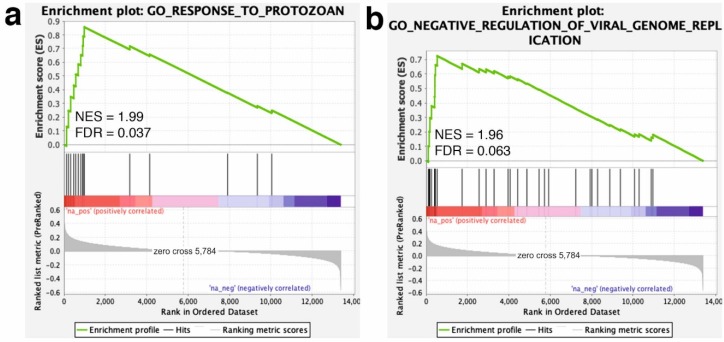
Gene set enrichment analysis (GSEA) plot (score curves) for enriched biological processes. The GSEA analysis was performed with the biological process gene sets of GSEA Molecular Signatures Database. The “Signal-to-Noise” ratio (SNR) statistic was used to rank the genes per their correlation with the dietary GP supplementation (red) or the un-supplemented CTR phenotype (blue). The heatmap on the right side of each panel visualize the genes mostly contributing to the enriched gene set. For the fully detailed list, see Supplementary Table S3. The green curve corresponds to the Enrichment Score (ES) curve, which is the running sum of the weighed ES obtained from the GSEA software, while the Normalized Enrichment Score (NES) and the corresponding *p*-value are reported within each graph. Panels (**a**,**b**) denote the most enriched (significant) biological processes (i.e., gene sets).

To further pinpoint the collective effect of the transcriptomic changes, we used GSEA to identify long-term GP-supplementation-associated pathways and biological processes. The GSEA analysis revealed that most of the core-enriched genes in the GP group, such as ‘interferon alpha (IFN-α) response’ (NES = 2.02), ‘IFN-γ response’ (NES = 1.94), ‘IL6-JAK-STAT3 signaling’ (NES = 1.74) or ‘complement’ hallmark genes (NES = 1.57), fell into the immune system-related hallmark gene sets (Figure 2). Moreover, some biological processes related with immune defense mechanisms were positively enriched in the GP group. Among these ones, the most enriched processes were the ‘response to protozoan’ (NES = 1.99) and the ‘negative regulation of viral replication’ (NES = 1.96; Figure 3). A summary of the GSEA analysis output is reported in Supplementary Table S3.

## 4. Discussion

The objective of this study was to examine the transcriptomic signature of a long-term dietary GP supplementation in dairy cows and to evaluate whether this signature reflects known GP-associated potential health benefits [37]. The main finding was that a 60-day dietary GP supplementation can modulate the expression of a considerable, albeit less than expected, number of genes in dairy cows and induce a noticeable transcriptomic signature that mainly reflects a GP-induced immunomodulatory effect.

Several studies assessed the effects of GP on milk production and composition in dairy cows. In two recent and independent studies, a dietary addition of ~10% GP (~2 kg GP/head/day for~60 days) did not show differences in milk composition [38,39]. Moreover, different concentrations (50, 75 and 100 g/kg of DM) of grape residue silage did not affect milk production nor the concentrations of CP, fat or lactose in dairy cows throughout a 21 days experimental period [10]. Further, the milk of dairy cows fed for three months with a diet containing 15% GP preserved the normal levels of fat, protein and caseins [22]. Finally, in a 4-week trial, dietary grape marc did not affect milk yield, milk protein or milk fat content of mid-lactation Holstein cows [40]. Overall, the present data further support the evidence that dietary GP does not alter milk yield or composition even after long-term supplementation.

Grape pomace accounts for ~25%–35% of the total weight of processed grapes; it contains significant amounts of dietary fiber, for which it may partially replace the forage portion in ruminants’ diet [22,37]. Far more interesting, GP is enriched with potent bioactive compounds, especially polyphenols (~2%–6.5%). Grape polyphenolics, possessing different chemical structures and activities, are essentially categorized into two major classes of compounds: flavonoids (the most abundant polyphenols) and non-flavonoids [37,39,41]. Even though the biological activity of GP extracts mostly relate to their antioxidant properties, the dietary intake of these bioactive compounds results in pleiotropic health-promoting responses, e.g., cardio- and neuroprotective, anti-diabetic, anti-cancer, anti-aging, anti-adipogenetic as well as prebiotic and anti-microbial beneficial effects [39,41,42,43]. Overall, these evidences would suggest a link between polyphenol-rich food consumption and reduction in the incidence of numerous chronic disorders effect [42]. A separate discussion deserves the effects of polyphenols on the immune system, which is the second most important mechanism of action (after the antioxidant activity). Several studies have shown how polyphenols target multiple inflammatory components and lead to anti-inflammatory mechanisms and/or inflammation antagonism. Further, polyphenols today represent also promising candidates for the therapy of autoimmune diseases [43,44,45,46]. 

In this study, the change in expression of more than 40 protein-coding genes indicates that dietary GP has a traceable transcriptomic signature. Grape pomace contains lipid, proteins, fiber, minerals and large amounts of polyphenols (e.g., 10.4–64.8 g gallic acid equivalents/kg); moreover, polyphenolics show a linear correlation with in vitro antioxidant activity [47]. Therefore, one can safely presume that present transcriptional changes are due to GP polyphenols content. However, in the present experimental condition, only a few DEGs were significantly modulated by the dietary GP supplementation, and no genes were markedly over-expressed (in terms of FC). This was not surprising as in transcriptomics (e.g., microarray, RNA-seq), a large part of biologically meaningful DEGs possess FC less than 2. As a whole, these DEGs are not a minor population; rather, a significant fraction of transcriptional changes occurring in that sample (tissue, blood, etc.) [48]. This begs the question of whether such small changes reflect biologically meaningful events. In nutrigenomic studies, nutritionally relevant concentrations of bioactive compounds may elicit subtle changes in gene expression, resulting in critically important biological insights even though difficult to be detected reliably [24,49,50,51]. Moreover, small transcriptional variations are somehow expected when investigating long-term effects of a supplemented diet. In our opinion, such an event should not be ignored. Noteworthy, some interesting genes such as DNAJA1, MFF, IMPACT, ABCA13, RGS2, and ITGA were significantly downregulated in animals fed with a GP-supplemented diet (FDR < 0.05).

The top downregulated gene, DNAJA1, also known as heat shock protein (HSP) 40, plays a fundamental role during both physiological and stress conditions as it functions in mediating inappropriate proteins aggregation and folding [52]. Several studies have suggested that some heat shock proteins (both HSP70 and HSP40) are induced by wide ranges of stressful and pathological conditions [53]. Intriguingly, the natural flavonoid quercetin, containing a polyphenolic chemical structure and possessing healthy antioxidant properties, was shown to inhibit the expression of some HSPs at basal levels in human tissues [54,55,56]. Therefore, we might suggest that a lower expression of DNAJA1 in the GP group of animals compared to controls could be associated to healthier conditions of cows fed a GP-supplemented diet.

In living organisms, mitochondria continually change their morphology to maintain cell homeostasis. This process (mitochondrial dynamics) consists of two interconnected events, i.e., fission and fusion [57,58]. Mitochondrial fission is mediated by dynamin-related protein 1 (Drp1), a cytosolic highly conserved guanosine triphosphatase of the dynamin superfamily of proteins [57,59,60]. In mammals, the recruitment of Drp1 to mitochondria is mediated by MFF, a member of an array of Drp1-binding receptor factors localized at the mitochondrial outer membrane [57,58,60,61]. Fission is a key mechanistic step in apoptosis induction. Furthermore, Drp1 and MFF may contribute to apoptotic phenomena resulting from oxidative stress and reactive oxygen species (ROS) overproduction [57,58,60,62,63]. Some xenobiotics trigger oxidative stress, mitochondrial disfunction and ultimately apoptosis by increasing the expression of Drp1 (mostly) and MFF; among these ones ammonia [63], T2-toxin [64], cadmium [65], and microcystin [66]. However, curcumin, resveratrol, anthocyanins, quercetin and polyphenols inhibit mitochondrial fission through transcriptional and post-translational modifications of Drp-1, MFF as well as of other factors contributing to mitochondrial dynamics, thus preventing cells from oxidative stress [67,68,69]. Therefore, present MFF downregulation might be viewed as a protective effect provoked by GP supplementation against cellular oxidative stress, resulting in minor recruitment and transfer of Drp-1 inside mitochondria and, consequently, no mitochondrial fission/fusion imbalance and apoptosis. Concomitant inhibition of Drp-1 (i.e., DNM1L) was expected but no effects were observed for this gene.

IMPACT is a protein-coding gene highly expressed in neuronal tissue of mammals, where it promotes neuronal development [70,71,72]. Noteworthily, it substantially contributes to the adaptive response to nutritional stress and dietary restriction (i.e., a reduction in food intake without malnutrition) [70]. Under these stress conditions (e.g., amino acids depletion, proteasome inhibition, lack of glucose), IMPACT can inhibit a serine/threonine kinase coded by the General Control Non-derepressible 2 (GCN2) gene [70,71,72,73,74]. This latter gene, which is not listed among the DEGs, confers an adaptive response through distinct biochemical reactions (e.g., phosphorylation) and stress response genes activation. This cascade of events constitutes the Integrated Stress Response (ISR) pathway [70]. In addition to GCN2 inhibition, IMPACT overexpression also abrogates the phosphorylation of another GCN2 molecular target, i.e., the eukaryotic initiation factor 2, α subunit (eIF2α) [70,73]. Interestingly, IMPACT, GCN2 and ISR “molecular sensors” contribute substantially to immune system regulation [71,72,74,75]. This crosstalk between nutrient metabolism, gut immune system and host microbiota potentially shapes the overall gut responses to nutrients, and today represents a “hot” research topic [76,77]. As to IMPACT, KO mice are leaner than wild-type ones, especially when fed a high-fat diet. Furthermore, they show a defect in the control body temperature in response to starvation. This evidence, albeit not completely independent from GCN2, would suggest that IMPACT protein is involved in the maintenance of energy homeostasis [72]. The IMPACT gene downregulation we observed in the present study might be hypothetically interpreted as the result of a positive effect of GP on cattle nutritional status and immune system.

RGS2 is a conserved regulator of G protein-coupled receptors (GPCRs) signaling pathway. It belongs to the RGS superfamily of proteins, whose expression is regulated by epigenetic, transcriptional and post-translational mechanisms. These proteins determine the intensity and duration of GPCR-mediated effects through binding to the active Gα subunit of G proteins and activating GTPase. This protein-coding gene is expressed in several tissues such as CNS, heart, vasculature, kidney, immune system, lungs, bone and ovaries [78,79]. Interestingly, oxidative stress conditions increase RGS2 mRNA levels [79,80]; moreover, RGS2 contributes to the regulation of some enzymes involved in antioxidant defense, namely glyoxalase-1 and glutathione reductase-1 [81]. Finally, RGS2 has been shown to be involved in the pathophysiology of gastrointestinal inflammation and visceral pain, mostly regulating T-cell immunity [79,82,83]. In our opinion, the observed RGS2 gene downregulation can be viewed as the consequence of an indirect positive effect of GP on the overall cattle gastrointestinal tract homeostasis.

Limited information is currently available for the remaining two DEGs, i.e., ABCA13 and ITGA3. The former is a member of the ATP-binding cassette (ABC) superfamily of efflux drug transporters, encoding for the largest protein of the same family (5,058 residues) [84]. This gene is expressed in trachea, testis, bone marrow and blood-derived cells [85,86]. Interestingly, broilers fed diets supplemented with trace minerals (Zn, Cu, and Mn) showed reduced footpad lesions and improved wound healing process. At the molecular level, upregulation of genes involved in collagen synthesis, deposition and organization, cell migration, matrix remodelling, and angiogenesis, including ITGA2 and ITGA3, was noticed [87]. Despite this, no information about a possible modulatory effect of polyphenols on ABCA13 and ITGA3 are actually available.

In the present study, most of the core-enriched genes in the GP group (e.g., IFN-α, IFN-γ, and IL6-JAK-STAT3) belong to hallmarks and biological processes related to the immune response. All this was expected. Widespread evidence shows that polyphenols possess predominantly anti-oxidant, anti-inflammatory and immunomodulatory properties in both humans and farm animal species including cattle; hence, they are beneficial all-purpose nutraceuticals or supplements [45,46,88]. Polyphenols inhibit NF-κB, PI3K/Akt, MAPKs, and arachidonic acid-dependent signaling pathways. Regarding their immunosuppressant properties, polyphenols modulate T-cell function (e.g., suppressing T helper 2 activation and promoting the development of regulatory T cells), inhibit mast cell degranulation, and downregulate inflammatory cytokine responses [45,46,76]. Owing to these immunomodulatory properties, polyphenols have emerged as potential tools for the treatment of autoimmune disorders [46,89].

Additional biological processes positively enriched in GP group were the ‘response to protozoan’ and the ‘negative regulation of viral replication’. Again, our results corroborate recent evidence about the use of polyphenolic compounds as potential therapeutic tools against protozoa and viruses, albeit scarce information is available on farm animals. Some dietary polyphenols (e.g., quercetin, resveratrol, rutin) show a certain efficacy against leishmaniosis [90], and giardiasis [91]; furthermore, resveratrol itself has been suggested as a potential restorative agent against toxoplasmosis brain disorders [92] and *Schistosoma mansoni* infections [93]. As far as the effects on viruses, human and animal evidence suggest polyphenolic compounds (e.g., resveratrol, tea polyphenols) possess potential preventive and therapeutic effects, and might be used as adjuvant therapy for the management of viral infection, e.g., influenza, herpes simplex, pseudorabies, hepatitis C, porcine reproductive and respiratory syndrome, and even Ebola [88,94,95,96,97].

## 5. Conclusions

This study provides evidence on the transcriptomic signature of long-term dietary GP supplementation in dairy cows. The present results strengthen the concept of using a nutrigenomic approach to assess the beneficial nor detrimental effects of farm animals’ diets supplemented with grape polyphenolics and, in general, with undervalued food-related by-products and sources such as aromatic plants, culinary herbs, spices, vegetables and fruit trimmings. In our experimental conditions, the GP dietary supplementation resulted in a differential transcriptomic signature mostly reflecting an immunomodulatory effect. Overall, these data contribute to the growing body of nutrigenomics research in dairy cows and, to a wider extent, in ruminants. 

Although additional studies should be performed to ascertain benefits and drawbacks of GP supplementation in dairy cows, and to define the optimal feeding schemes, the results here obtained let us suggest that this agricultural by-product has an overall positive impact on immune status of cows, without affecting milk quality. 

The present study comes with some shortcomings. First, the number of DEGs was lower than expected; moreover, most of them were downregulated and showed lower FC. Nevertheless, we were expecting to find a higher number of DEGs, possibly confirming (e.g., gene upregulation) the antioxidant and anti-inflammatory properties of grape polyphenolics. Actually, the transcriptomic signature of GP-supplemented animals (namely, the list of DEGs and GSEA outputs) is suggestive of improved dairy cows’ general health conditions, thereby confirming the GP wide range of health-promoting activities, e.g., anti-inflammatory, anti-aging, anti-oxidant, anti-microbial and antiviral properties. Moreover, it is important to highlight that feed might exert its biological effect at different levels. Thus, it would be possible that GP targets mechanisms other than transcription, such as mRNA silencing, mRNA translation, and enzyme activity/functionality. To this respect, the previous literature demonstrated that GP supplementation affects catalase activity in lambs [18], superoxide dismutase and glutathione peroxidase activity in piglets [98]. To our knowledge, proteomic studies investigating the effect of GP have not been so far performed in farmed animals. However, it is likely that protein expression may be a target of GP. Indeed, dietary inclusion of other bioactive components have been demonstrated to significantly affect the intestinal mucosa proteome of growing pigs [99] and the skeletal muscle proteins of pre-weaning calves [100].

Another possible limitation of the study is having measured the GP-dependent transcriptomic changes in dairy cows WB. Many experiments aiming at the characterization of the beneficial effect of nutraceuticals on the individual’s transcriptome involve the use of gastrointestinal biopsies (e.g., in rodents, chicken, fish). Apart from the ethical considerations, there is evidence that the WB is a suitable surrogate tissue for transcriptomics studies. The final potential bias is intrinsic to grape polyphenolics composition and the corresponding biological activity. In general, the varying composition of GP originating from different grapes and locations are likely to show remarkable differences in their biological activity. As a consequence, this “biodiversity” impacts GP bioavailability, metabolism, bioactivity and, ultimately the animal’s transcriptomics signature. This is still a major criticism of the use of grape polyphenols in food-producing animals as well as in other species including humans. In perspective, crude or highly purified forms of GP extract might be used to characterize the most of beneficial effect of a GP-supplemented diet. Despite these potential biases, the present results corroborate the health benefits, sustainability, and environmental impact of GP utilization in dairy animals and in general in food-producing species. Clearly, further investigations are recommended to improve our knowledge on the best use of polyphenolic compounds as nutraceuticals, e.g., evaluating different feeding schemes and/or percentage of GP supplementation, exploring the now in fashion “host-drug-nutraceutical-microbiota interactions”.

## Figures and Tables

**Table 1:** Chemical composition and main total polyphenols content of the GP extract.

Chemical Composition of GP	
Dry Matter, %	92.20
Crude protein ^1^, %	12.40
Ether extract ^1^, %	4.20
Crude fibre ^1^, %	31.80
Neutral-detergent fibre ^1^, %	42.20
Acid detergent fibre ^1^, %	41.30
Acid-detergent lignin ^1^, %	28.80
Starch ^1^, %	-
Ash ^1^, %	8.50
Total Polyphenols Content (TCP) in GP	
TPC (GAE ^2^ mg/g)	16.07 ± 1.41

^1^ On a dry matter (DM) basis; ^2^ Gallic Acid Equivalents.

**Table 2:** Ingredients and chemical composition of the two diets.

	Diets	
Ingredients of the diets	CTR/basal	GP
Corn silage, %	45.70	45.50
Second-cut alfalfa hay, %	20.30	20.20
Wheat straw, %	3.80	1.30
Corn grain meal, %	13.50	13.10
Soybean meal, %	8.40	7.60
Barley meal, %	2.50	2.50
Cotton seed, %	3.80	2.80
Dried grape pomace, %	-	5.00
Vitamins and minerals, %	2.00	2.00
Nutrient composition		
Dry Matter, %	61.50	61.70
Crude protein ^1^, %	15.30	15.90
Ether extract ^1^, %	2.30	2.20
Crude fibre ^1^, %	17.80	18.90
Neutral-detergent fibre ^1^, %	34.40	36.30
Acid detergent fibre ^1^, %	20.30	22.50
Acid-detergent lignin ^1^, %	4.60	5.90
Starch ^1^, %	25.20	24.80
Ash ^1^, %	4.50	5.10
Net energy (Milk UF/kg DM)	0.89	0.91

^1^ On a dry matter (DM) basis.

## Supplementary Materials

The following are available online at https://www.mdpi.com/2076-2615/10/4/714/s1. Figure S1. Effects of dietary GP supplementation on milk production. Table S1. Sequencing and alignment summary. Table S2. Differentially Expressed Genes (DEGs). Table S3. Summary of the GSEA analysis output.
