# Supplementary figures and images for: Nutrigenomic Effects of Long-Term Grape Pomace Supplementation in Dairy Cows
Source: Animals (Basel). 2020 Apr 19;10(4):714. doi: 10.3390/ani10040714 (PMC7222749; doi:10.3390/ani10040714)

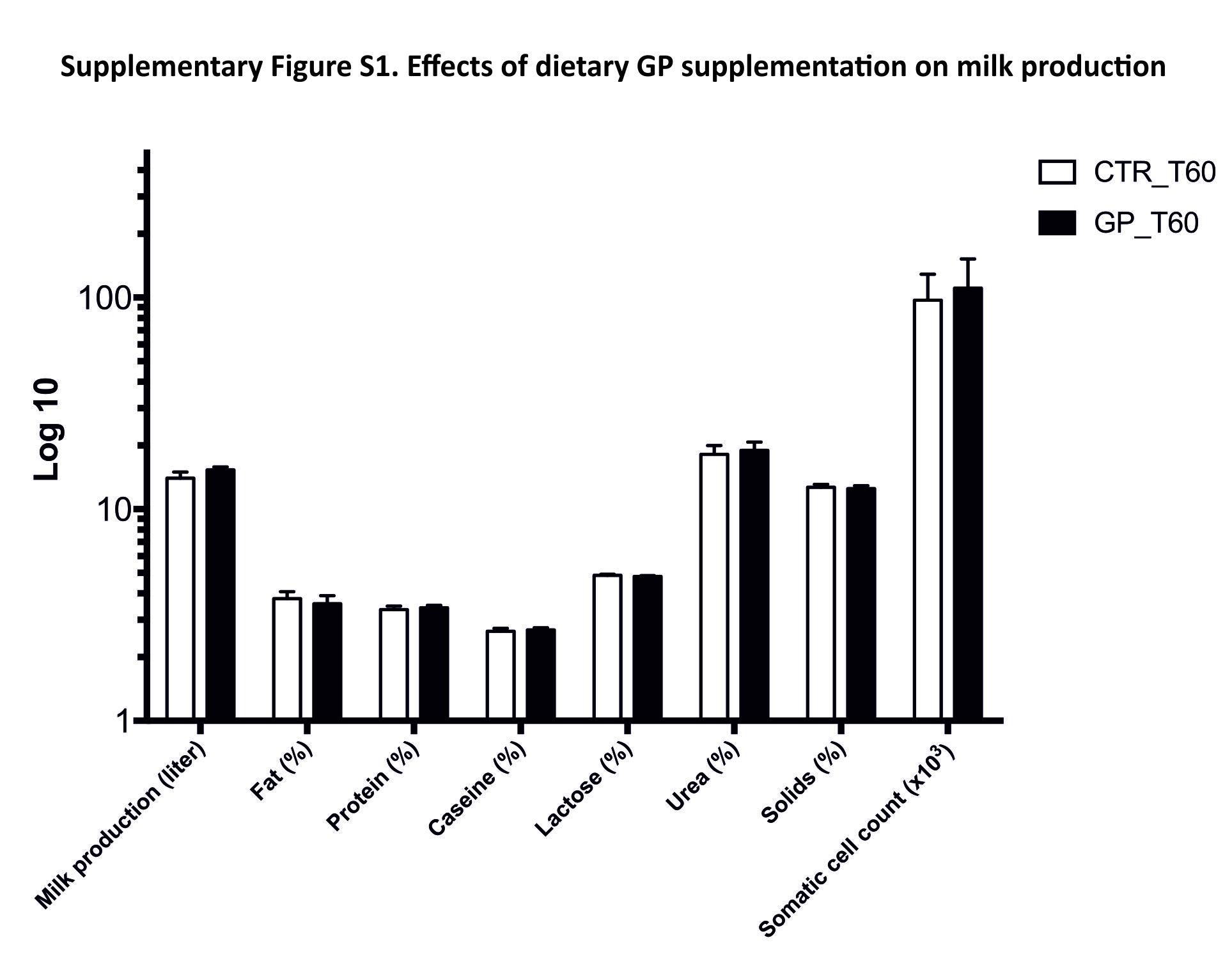

Supplement: Supplementary file 1 [file animals-10-00714-s001.zip › Supplementary Material REVISED/Supplementary_FigureS1.jpg]
